# Effect of Tributyrin on Growth Performance and Pathway by which Tributyrin Regulates Oligopeptide Transporter 1 in Juvenile Grass Carp (*Ctenopharyngodon idellus*)

**DOI:** 10.3390/ani12192498

**Published:** 2022-09-20

**Authors:** Zhimin He, Na Liu, Yuyang Cai, Na Yang, Gen Li, Yang Xiao, Xiaomei Zhou, Shenping Cao, Fufa Qu, Jianzhou Tang, Suchun Liu, Zhen Liu

**Affiliations:** 1 Hunan Provincial Key Laboratory of Nutrition and Quality Control of Aquatic Animals, Department of Biological and Environmental Engineering, Changsha University, Changsha 410022, China; 2 College of Food Science and Technology, Hunan Agricultural University, Changsha 410128, China

**Keywords:** CDX2, grass carp, gene expression, growth performance, PepT1, regulation pathway, SP1, tributyrin

## Abstract

**Simple Summary:**

Oligopeptide transporter 1 (PepT1) plays a role in the transportation and absorption of oligopeptides, which is an important part of protein nutrition and affects the growth of animals. Tributyrin (TB), the precursor of butyrate, exhibits similar functions to those of the butyrate in intestinal nutrients absorption. The analysis of TB on the growth of grass carp and its regulation pathway on PepT1 may help us to better understand the functions of TB and oligopeptide transportation via PepT1, which can be modulated by diet. In this study, we demonstrated that an appropriate level of tributyrin supplementation in the diet promoted the growth of juvenile grass carp and elevated the expressions of caudal type homeobox 2 (CDX2), specificity protein 1 (SP1), and PepT1 in the grass carp intestine and primary intestine cell. In addition, CDX2 and SP1 regulating the expression of PepT1 was investigated. Finally, CDX2/SP1-mediating tributyrin regulation on PepT1 was elucidated. This study verified the effect of tributyrin on the growth of juvenile grass carp and clarified the tributyrin regulation pathway on CDX2/SP1-PepT1.

**Abstract:**

The nutritional functions of tributyrin (TB) have been extensively studied, but questions remain regarding its influence on the growth of juvenile grass carp (*Ctenopharyngodon idellus*) and the regulation pathway to PepT1 in the intestine of grass carp. To answer the remaining questions, feeding trials, cell trials, and peritoneal injection trials were conducted in this study. The results showed that an appropriate level of TB (0.5 g/kg and 1.0 g/kg) supplementation in feed significantly promoted the growth performance of juvenile grass carp. The expressions of intestine genes (CDX2, SP1 and PepT1) related to oligopeptide transportation increased in the 0.5 g/kg TB group of feeding trials and both the 5 mM and 10 mM TB groups of the intestine cell trials, respectively. Subsequently, the injection trials of inhibitors CDX2 and SP1 demonstrated that the inhibition of CDX2 or SP1 decreased the mRNA expression of PepT1. Finally, the results of independent or combined treatments of TB and the inhibitors suggested that CDX2/SP1 mediated TB regulation on PepT1. These findings may help us to better understand the functions of TB on growth and PepT1 oligopeptide transportation, which could be modulated by dietary TB through the CDX2/SP1-PepT1 pathway in juvenile grass carp.

## 1. Introduction

Tributyrin (TB) is a triglyceride naturally present in butter. It is an ester composed of butyric acid and glycerol that can act as a precursor of butyrate as it can be cleaved to butyrate by intracellular enzymes [1,2,3]. Butyrate and its salts as feed additives have been found to play important roles in intestinal nutrient absorption and health and were considered as a potential substitute of antibiotic feed additives [4,5,6,7]. In terms of aquatic animals, appropriate levels of sodium butyrate can be a desirable growth promoter to stimulate growth and nutrient retention in juvenile yellow catfish [8]. Sodium butyrate combined with lippia origanoide essential oil supplemented in the commercial diet of *Nile tilapia* juveniles can reduce the concentration of total heterotrophic bacteria in the intestine [9]. In addition, sodium butyrate nanoparticles can boost the antioxidant status and immunity of *Oreochromis niloticus* [10]. Although it has several advantages for the growth of aquatic animals, its characteristics such as volatility, special odor, and instability obstructed its application in aquatic production [11,12]. However, the precursor of butyrate, tributyrin, could make up some shortcomings of butyrate and its salt. Compared with the butyrate, the maximum advantage of tributyrin is that it could smoothly pass through the stomach without being decomposed by gastric acid and work as a butyrate sustained-release agent in the back end of the intestine to promote nutrition absorption and protect gut health [13,14,15].

Until now, tributyrin was primarily used as a feed additive to improve intestinal function, growth performance, immunity, intestinal flora, and the meat quality of mammals or poultry. Tributyrin can significantly improve the feed intake and daily weight of weaned piglets, reduce piglets’ diarrhea, improve the intestinal morphological structure, and alter intestinal microbiota and metabolites [16,17,18,19]. Dietary supplementation with tributyrin and glycerol monolaurate has been shown to improve the growth performance and feed conversion and change the rumen microbiome of weaned lambs [20]. Meanwhile, it also improved intestinal morphology and influenced the short-chain fatty acid profiles in broilers [21,22]. Its functions to promote the growth of yellow-feathered broilers and improve the performance of broiler chickens during Eimeria maxima-induced coccidiosis were demonstrated [23,24]. Additionally, tributyrin stimulated growth, decreased the urea blood concentration, and elevated the serum insulin concentration of weaned piglets [18]. Moreover, the dietary inclusion of tributyrin improved the reproductive parameters, egg quality, intestinal morphology, and antioxidant capacity of breeders [25,26,27]. Although its application is rare in aquaculture, it was still evidenced that tributyrin supplemented in the feed of juvenile Blunt Snout Bream (*Megalobrama amblycephala*) promoted growth performance and health status [28].

The intestine-specific gene CDX2 has been investigated to be stimulated by sodium butyrate at mRNA and protein levels in human adenocarcinoma cell lines Caco2 and HT29 [29]. The butyrate was demonstrated to improve the binding activity of CDX2 to the PepT1 promoter to increase the mRNA expression of PepT1, and PepT1 transport activity enhanced in mouse colonic apical membranes vesicles with 5 mM butyrate added to mouse drinking water for 24 h [30]. CDX2 was also elucidated to be a potential factor to mediate the regulation of sodium butyrate on PepT1 in ploidy fishes [31]. Tributyrin/butyrate enabled the interaction between SP1 and p300 to facilitate p300-HAT binding [32]. Additionally, SP1 was evidenced to mediate butyrate or sodium butyrate regulation on several genes. For instance, sodium butyrate induced the transcription of Claudin-1 or G alpha(i2) by facilitating the interactions between SP1 and their promoters, respectively [33,34]. In addition, butyrate decreased the binding affinity of SP1 to the Neuropilin-1 (NRP-1) promoter to decline NRP1 expression [35]. Sodium butyrate treatments increased PepT1 expression in the intestine of grass carp in vivo and in vitro [36]. Furthermore, CDX2 was investigated to positively regulate PepT1 through SP1 as no CDX2 binding sites exist in the human PepT1 promoter, while it has binding sites of SP1. CDX2 interacted with SP1 and could not regulate the expression of PepT1 when the SP1 binding site on PepT1 was mutated [37], while CDX2 was investigated to bind the PepT1 promoter directly to regulate its transcription activity in grass carp [38]. According to the reference studies, the intestine genes CDX2, SP1, and PepT1 were regulated by butyrate, sodium butyrate, and tributyrin, while the tributyrin regulation pathway on PepT1 remains elusive.

The present study aims to investigate the effect of TB on the growth of juvenile grass carp and the gene expressions of the oligopeptide-transportation-related intestinal genes of juvenile grass carp fed a diet with different levels of tributyrin; optimize the supplementation level of TB in juvenile grass carp feed; and identify the roles of TB in the oligopeptide transportation pathway. This study seeks to update TB application in grass carp feed and clarify TB regulation pathway to PepT1 through experiments.

## 2. Materials and Methods

### 2.1. Experimental Diet

Four isonitrogenous (300 g crude protein per kg diet), isolipidic (80 g crude lipid per kg diet), and isocaloric (19 MJ/kg diet) diets were formulated with tributyrin (TB, purity ≥ 99%, Guangdong Yiduoli Co., Ltd., Zhuhai, China) and added at the levels of 0.0, 0.5, 1.0, and 1.5 g/kg diet, while 0.0 g/kg tributyrin was the control group. The diet formulation and chemical composition are shown in Table 1. Protein sources were provided by fish meal, and soybean meal and lipid sources were supplied by fish oil and soybean oil, with calcium dihydrogen phosphate as a phosphorus source. The raw materials were ground separately and passed through a 40-mesh sieve (0.425 mm diameter). Subsequently, the sieved raw materials were mixed with the lipid source such as soybean oil and water with the addition order of pellet size from small to large in a mixer (model: CH-100, Jiangyin, China). After being mixed thoroughly, the mixture was extruded into 1.5 mm pellets by a laboratory granulator (SZLH200, Jiangsu Zheng chang Group Co. Ltd., Changzhou, China) and placed plainly on the ground out of the door to air dry. The prepared pellets were finally stored in separate sealed plastic bags at −20 °C until use.

### 2.2. Feeding Experiment Design

Healthy juvenile grass carp that were hatched in the same batch were purchased from the Hunan Institute of Aquatic Science. Prior to the experiment, the experimental fish were fed daily with a standard diet (2 mm diameters) for 1 week in a room recirculating water tank system with continuous aeration at 24–27 °C, and the water pH was monitored to keep it between 6.5 and 7.5 to adapt to the culture environment and experimental feed. A four-week feeding growth experiment was subsequently implemented to analyze the influence of different levels of tributyrin on grass carp. A total of 320 fish (43.50 ± 0.5 g) with uniform size were stochastically selected and randomly assigned to 16 tanks (0.3 m^3^). Twenty grass carps were put into each tank for per treatment with four repetitions (*n* = 4 × 20 fish/treatment). The experimental feed was given during the experiment period 3 times per day (at 9:00, 12:00 and 18:00). The oxygen aeration was maintained for day and night, and the temperature and pH were kept 24–27 °C and 6.5–7.5, respectively.

Feeding was stopped one day prior to the end of feeding experiment. The weight of live fish in each tank was weighed, and the number was counted. Then, calculations were performed according to the following equations: average weight (g/fish) = total weight (g)/number of fish; survival rate (SR, %) = (final number/initial number) × 100%; weight gain rate (WGR, %) = (final average weight-initial average weight)/initial weight × 100%; specific growth rate (SGR, %) = [ln (final average weight) − ln (initial average weight)]∕number of days × 100%.

### 2.3. Sample Collection

The experimental fish were fasted for 24 h after the 4-week experimental feeding period, and 3 fish were randomly taken from each tank (*n* = 4 × 3 fish/treatment). The collected fish were dissected to separate out the whole intestine and scratch out the intestinal content with scissors and a blade, which was then washed in PBS buffer 3 times, and finally the liquid on the intestine was blotted with filter paper. Subsequently, the whole intestine was cut into pieces with sterile scissors and put into a 1.5 mL RNase free sample collection tube (Axygen, MCT-150-C-S, Union City, CA, USA). Finally, samples were immediately frozen in liquid nitrogen and stored at −80 °C for use.

### 2.4. Grass Carp Intestinal Cell Culture

To examine the effect of tributyrin on the mRNA levels of CDX2, SP1, and PepT1 in vitro, primary cell cultures of grass carp intestines were performed according to previous research [39]. Five juvenile grass carp (50 ± 5.0 g) were acclimatized and maintained to be fasted in a tank with 24–27 °C water that contained 2% antibiotics (200,000 IU/L penicillin and 200 mg/L streptomycin) as well as continuous aeration for 1–2 days to clean up the intestine. The fish bodies were soaked in 0.01% potassium permanganate solution for 0.5 h to disinfect. After that, several drops of 2-phenoxyethanol were added to anesthetize the fish. The anesthetized fish were taken out from the potassium permanganate solution and sprayed with 75% ethanol to clean their body surface. Next, their small intestines were rapidly separated using scissors in biological safety cabinets. After washing three times with PBS containing 2% antibiotics (penicillin and streptomycin), the small intestine was longitudinally cut and soaked in 2% antibiotic (penicillin and streptomycin) solution for 10 min. Then, the small intestinal mucosa was scraped from the inner wall of the small intestine with a scalpel into a 1.5 mL sterile tube (Axygen) and sheared into small pieces as much as possible with the scissors. The sheared intestinal mucosa was pre-sterilized by 75% ethanol, diluted by 0.5 mL DPBS solution with 1% antibiotics (penicillin and streptomycin), and finally centrifuged for 3 min at 800 rpm/min. The sheared intestinal mucosa was washed with 3 mL DPBS solution containing 1% antibiotics (penicillin and streptomycin) and centrifuged for 5 min at 800 rpm/min three times. Subsequently, the treated intestinal mucosa was transferred to a 1.5 mL sterilized tube and incubated with 3 mL 0.25% trypsin at 28 °C for 10 min. The cell suspension was collected and centrifuged at 800 rpm/min for 5 min. The cell precipitate was washed with DPBS twice and diluted with 2 mL of Dulbecco’s Modified Eagle Medium (DMEM, Gibco, 12,100,046, Waltham, MA, USA). An amount of 200 μL of cell suspension was cultured in a 6-well culture plate (Corning Inc., I0905-187, Corning, NW, USA) with 2 mL of basal medium (DMEM) in an incubator at 28 °C with 5% CO_2_. The medium was replaced with fresh medium containing 10% fetal bovine serum (FBS, Gibco, 26140-079, MA, USA) after cell adherence; the medium was changed every 2 days until the cells had adhered well and were normally extended. Then, tributyrin was supplemented to the fresh medium to treat the cells. To examine the response to tributyrin in vitro, five different tributyrin concentrations (0 mM, 5 mM, 10 mM, 15 mM, and 20 mM) were tested with four replicates for each concentration. After 12 h of tributyrin treatment, cells were harvested and frozen in liquid nitrogen and stored at −80 °C for use. Each experiment was performed in triplicate.

### 2.5. Intraperitoneal Injection Trials

Healthy juvenile grass carp of uniform size and weight (about 25 ± 3 g) were purchased from the Hunan Institute of Aquatic Science (Changsha, Hunan, China). Before intraperitoneal injection, the grass carp were placed into an indoor tank (0.3 m^3^) with normal culture conditions (continuous aeration at 24–27 °C, pH 6–7) to adapt to the culture conditions for 3 days. The CDX2 inhibitor GS (CAS^#^: 39025-23-5, Yuanye, B290139, Shanghai, China) and SP1 inhibitor MA (CAS^#^: 18378-89-7, Yuanye, S17074, Shanghai, China) were injected into grass carp at different concentration (GS: 0 μM, 50 μM, 100 μM, 200 μM, and 500 μM; MA: 0 μM, 10 μM, 25 μM, 50 μM, and 100 μM, respectively) and at different times (0 h, 1 h, 3 h, 6 h, 12 h, and 24 h) to analyze the optimum treating concentration and time. Each injection treatment was administered to three fish with three replicates (*n* = 3 × 3 fish/treatment).

Based on the above injection trial, the optimum concentrations of GS and MA were used in combination with tributyrin, respectively, for another injection trial to analyze whether CDX2 or SP1 mediated the regulation of tributyrin on PepT1. Six groups (control (PBS solution of DMSO), 200 μM GS, 200 μM GS + 50 mM TB, 50 mM TB, 50 μM MA, and 50 mM TB + 50 μM MA) were set. Each group had three replicates with five fish. A total of 90 fish were used and divided into 18 tanks in this injection trial (*n* = 3 × 5 fish/group). After injection for 3 h, the whole intestines of three grass carp in each tank were collected and frozen in liquid nitrogen. The frozen intestine samples were transferred and stored at −80 °C until further analysis. 

### 2.6. RNA Extraction and cDNA Synthesis

The RNA was extracted by RNAiso Plus (TaKaRa, Kusatsu, Japan,) from the intestines of tributyrin feeding fish, intestinal cells treated with tributyrin, and intraperitoneal injection fish intestines, respectively. The first-strand cDNA synthesis was performed using 1 μg total RNA with a First-Strand cDNA Synthesis Kit (TaKaRa PrimeScript™ RT reagent Kit with gDNA Eraser, Kusatsu, Japan).

### 2.7. Quantitative Real Time PCR

The mRNA expression levels of CDX2, SP1, NF-κB, and PepT1 were determined by quantitative real-time PCR using a Thermo Fisher-Q3 Fluorescence quantitative PCR instrument. β-Actin, whose primer sequences have been proven to be suitable for grass carp mRNA expression analysis, was used as the internal control in this study [39]. For each sample, three replicates were performed under the following conditions: 95 °C for 30 s followed by 40 cycles at 95 °C for 10 s, 60 °C for 30 s. Relative mRNA expression was calculated using the 2^−ΔΔCt^ method in excel. The primers used in this study are designed with Primer Express software and shown in Table 2.

### 2.8. Statistical Analysis

All the results are shown as the mean ± SD of different biological samples. The data were firstly subjected to normality and homoscedasticity tests, and then statistical evaluation with one-way analysis of variance (ANOVA) was performed, which was followed by the Tukey multiple comparison test using SPSS 18.0. Different lowercase letters indicate significant differences (*p* < 0.05).

### 2.9. Ethics Statement

The research was monitored and listed under the guidelines of the relevant institutional animal care and guidance committee for all projects involving animal work. All of the experimental procedures applied in this study complied with the ARRIVE guidelines; were carried out in accordance with the U.K. Animals (Scientific Procedures) Act, 1986, and associated guidelines; and were approved by the Institutional Animal Care and Use Committee of Changsha University (Changsha, China).

## 3. Results

### 3.1. Appropriate Tributyrin Supplementation Promotes the Growth of Grass Carp

Tributyrin influence on the growth performance of grass carp with five indicators, including initial body weight (IBW), final body weight (FBW), survival rate (SR), weight gain rate (WGR), and specific growth rate (SGR) via the feeding experiment, was firstly determined. The growth performance results are summarized in Table 3. The SR of all diet treatment was 100%; the FBW, WGR, and SGR were statistically different among all groups. The treatment groups fed with 0.5 g/kg and 1.0 g/kg tributyrin inclusion in diet displayed significantly higher FBW, WGR, and SGR levels compared with the control group. The 0.5 g/kg and 1.0 g/kg tributyrin groups presented 9.89% and 22.04% higher FBW, 13.76% and 31.49% higher WGR, and 0.16% and 0.35% higher SGR, respectively, in comparison with the control group. The grass carp that received 1.5 g/kg of tributyrin supplementation feed showed no significant difference in FBW, WGR, and SGR with the control group. The above results illustrate that tributyrin has the function of promoting the growth of grass carp at appropriate levels.

### 3.2. Tributyrin Regulates the Expressions of CDX2, SP1, and PepT1 In Vivo and In Vitro

**Figure 1:**
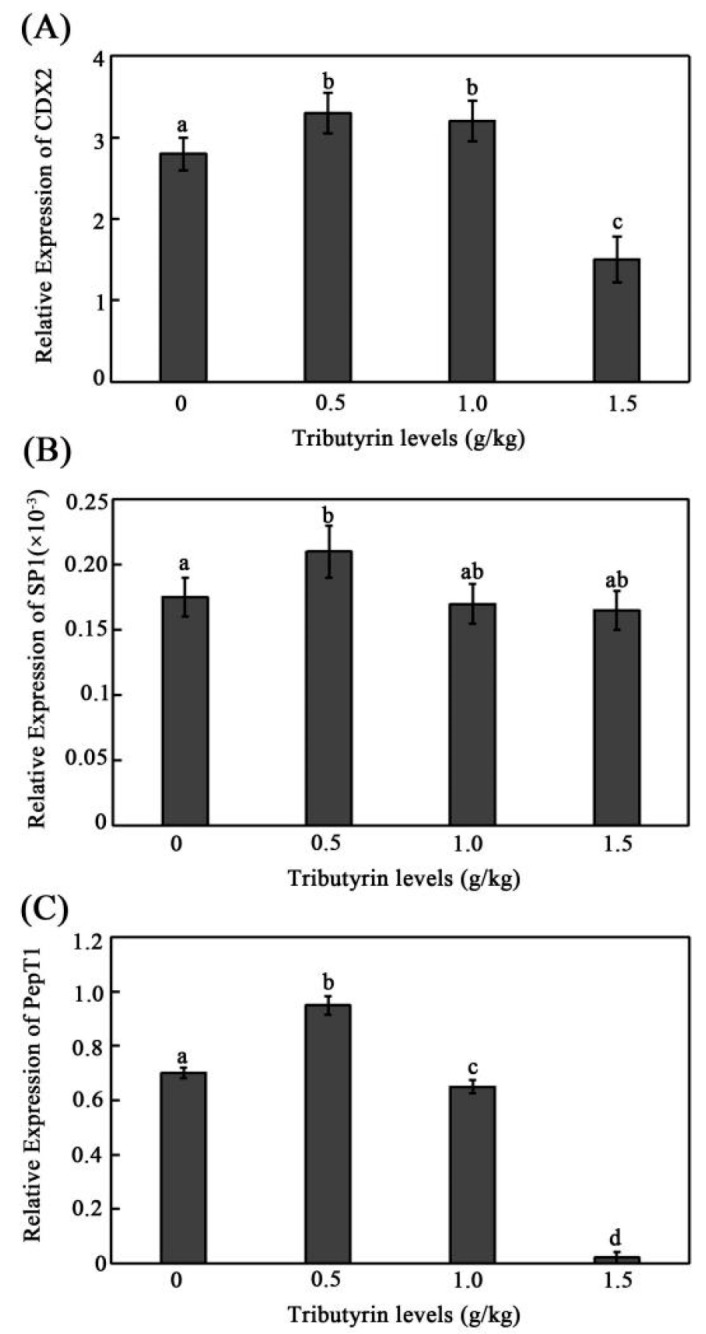
Effect of different levels of tributyrin supplementation in feed on the mRNA expression levels of grass carp intestinal genes. (**A**–**C**) The mRNA expression of grass carp intestinal genes CDX2 (**A**), SP1 (**B**) and PepT1 (**C**). The data represent the mean ± SD. Tukey’s multiple range test was performed, and different letters represent significant differences (*p* < 0.05, *n* = 4 for each treatment with a total of 12 fish).

To examine whether tributyrin regulates grass carp CDX2, SP1, and PepT1 expression, the mRNA expressions levels of CDX2, SP1, and PepT1 were analyzed in vivo and in vitro. Their expression levels increased along with the addition level of tributyrin and then declined (Figure 1). Compared with the control group, their expression levels reached their peak in the 0.5 g/kg treatment group and dropped off to the lowest in the 1.5 g/kg treatment group. In addition, the expression level of SP1 exhibited a significant difference only in the group of 0.5 g/kg compared with the control group (Figure 1B). These results may help to illustrate the tributyrin-promoting function in the growth of juvenile grass carp at an appropriate level through its stimulation on CDX2 or SP1 expressions and subsequently elevating the expression of PepT1, which would finally facilitate the uptake of peptides to improve the growth of grass carp. However, establishing whether CDX2 or SP1 plays a key role in the regulation of PepT1 may require further study.

**Figure 2:**
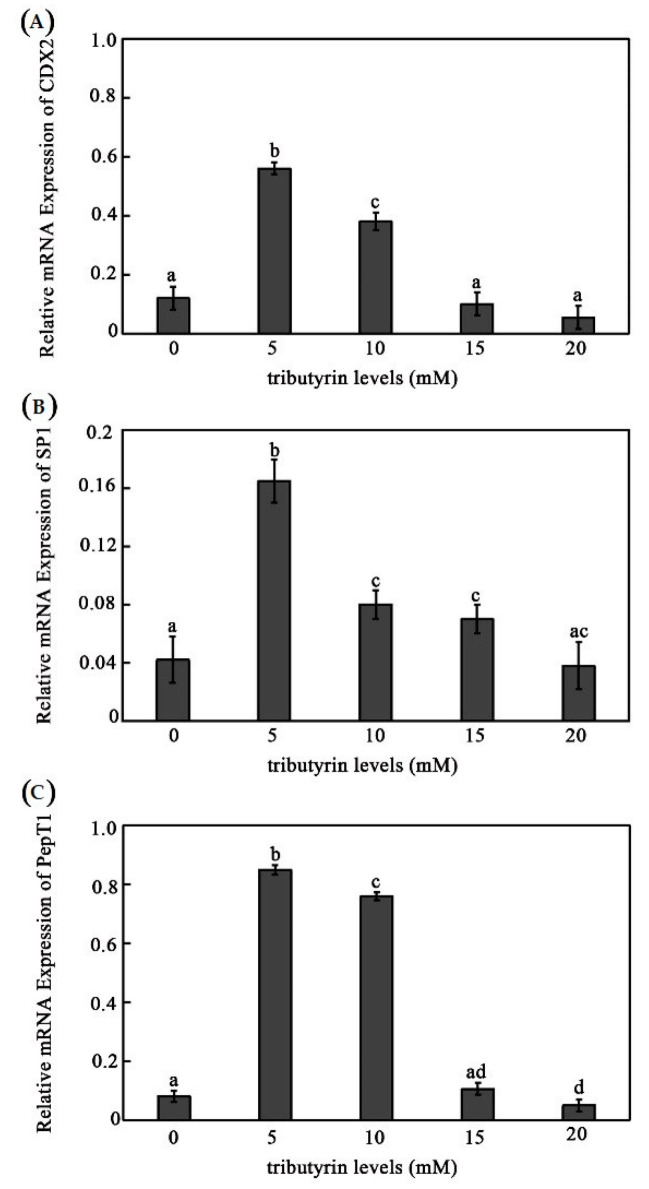
The influence of different concentrations of tributyrin on the expressions of CDX2, SP1, and PepT1 in the intestine cells of grass carp. (**A**–**C**) The mRNA expressions of grass carp intestinal genes CDX2 (**A**), SP1 (**B**) and PepT1 (**C**). The data represent the mean ± SD. Tukey’s multiple range test was performed, and different letters represent significant differences (*p* < 0.05, *n* = 4 for each treatment with three replicates).

We further examined the regulation of tributyrin on CDX2, SP1, and PepT1 in the grass carp intestinal cells. We found that all of them showed the highest expression level with the treatment of 5 mM tributyrin and then began to fall off. The expression change trends of CDX2, SP1, and PepT1 in grass carp intestine cells were similar with those in vivo. The expression levels of CDX2 and SP1 displayed no significant differences, and PepT1 was inhibited in the group of 20 mM tributyrin compared with those of the control group (Figure 2).

The results of tributyrin influence on the growth of juvenile grass carp and its regulation on CDX2, SP1, and PepT1 both in vivo and in vitro demonstrate that tributyrin could promote the growth of juvenile grass carp and increase the mRNA expressions of CDX2, SP1, and PepT1 at an appropriate level.

### 3.3. Effects of GS on Intestinal NF-KB and CDX2 Expression In Vivo

**Figure 3:**
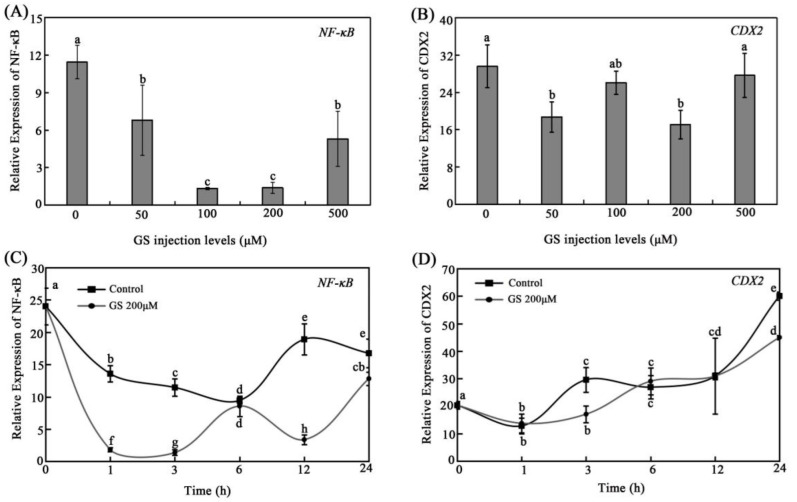
The effect of GS on the expression of NF-κB and CDX2 in intestines of grass carp. (**A**,**B**) The relative mRNA expression level of NF-κB (**A**) and CDX2 (**B**) of grass carp intestine with injection of different levels of GS. (**C**,**D**) The relative mRNA expression of NF-κB (**C**) and CDX2 (**D**) with 200 μM GS injection for different times. The data represent the mean ± SD. Tukey’s multiple range test was performed, and different letters represent significant differences (*p* < 0.05, *n* = 3 for each treatment with a total of nine fish).

Guggulsterone (GS) as a direct inhibitor of NF-κB has been evidenced to suppress the NF-κB-dependent activation of CDX2 expression [40]. To investigate the GS inhibition effect on grass carp gene expression, the mRNA expression levels of NF-κB and CDX2 were examined by qRT-PCR in vivo after treating grass carp with different concentrations of GS at different times. The results showed that different concentrations of GS had an obvious inhibition effect on NF-κB expression, especially the treating concentrations of 100 μM and 200 μM (Figure 3A). In addition, the mRNA expression level of CDX2 was significantly inhibited in the 50 μM and 200 μM GS treatment groups, while its expression had no obvious change with the treatment of 500 μM GS compared with the control group (Figure 3B). Here, 200 μM GS was selected for the subsequent GS injection trial for different treatment times due to its strong inhibitory effect on the expression of CDX2 among all applied GS concentrations. Furthermore, the results of the 200 μM GS injection trial showed that the expression level of NF-κB was significantly inhibited during the experimental period compared with that of the control group, except at the time point of 6 h (Figure 3C). The results showed that the expression level of CDX2 was inhibited at 3 h and 24 h post injection, with significant differences compared with the control group (Figure 3D).

### 3.4. Effects of MA on Intestinal SP1 Expression In Vivo

**Figure 4:**
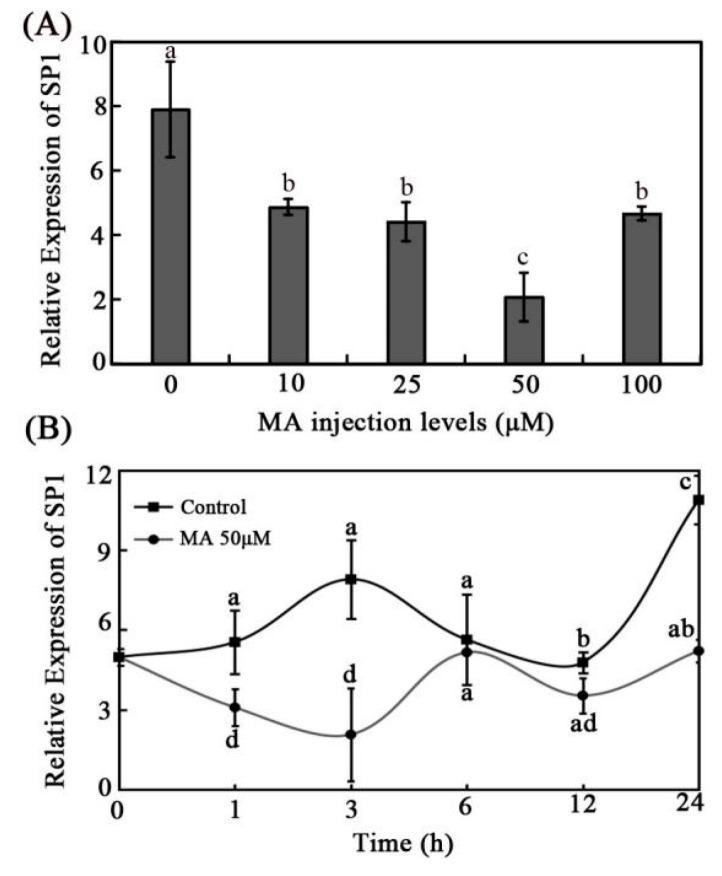
The effect of MA (SP1 inhibitor) on the expression of SP1 in intestines of grass carp. (**A**) The relative mRNA expression of SP1 with different levels of MA injection. (**B**) The relative mRNA expression of SP1 with 50 μM MA injection for different time. The data represent the mean ± SD. Tukey’s multiple range test was performed, and different letters represent significant differences (*p* < 0.05, *n* = 3 for each treatment with three replicates).

MA could inhibit the expression of SP1 [41]. To examine whether MA could inhibit the expression of intestinal SP1 of grass carp, the MA injection trial was implemented. The results demonstrated that the SP1 expression level was obviously decreased by different concentrations of MA, especially the 50 μM MA (Figure 4A). The results of different treatment times with 50 μM MA showed that the SP1 expression level was inhibited during the experimental period with a significant difference, except at 6 h post injection compared with the control group. Moreover, SP1 expression level decreased to the lowest level at 3 h post injection (Figure 4B).

### 3.5. The Analysis of CDX2/SP1 Mediating the Regulation of Tributyrin on the Expression PepT1

**Figure 5:**
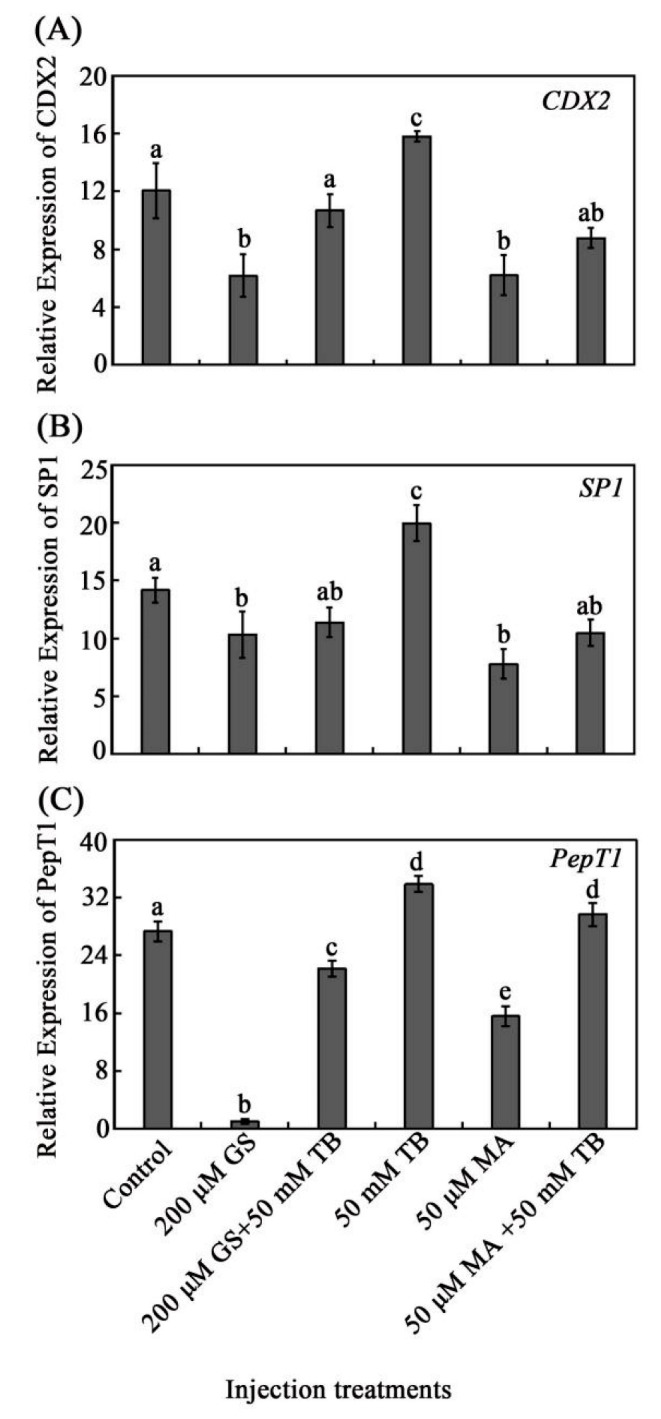
CDX2/SP1-mediated regulation of PepT1 by tributyrin. (**A**) The effect of indicated injection treatments on mRNA expression level of CDX2. (**B**) The effect of indicated injection treatments on mRNA expression level of SP1. (**C**) The effect of indicated injection treatments on mRNA expression levels of PepT1. The data represent the mean ± SD. Tukey’s multiple range test was performed, and different letters represent significant differences (*p* < 0.05, *n* = 3 for each group with a total of 15 fish).

To investigate whether CDX2 or SP1 mediated the regulation of PepT1 by tributyrin, TB was injected alone or in combination with GS or MA in the injection trials. The results suggested that CDX2, SP1, and PepT1 expressions were elevated with the treatment of tributyrin and inhibited by both GS and MA when compared with the control. The expressions of CDX2, SP1, and PepT1 that were boosted by tributyrin were declined by GS and MA. Additionally, their expression would be partially recovered when tributyrin was injected together with GS or MA (Figure 5). These results demonstrated that tributyrin regulated CDX2/SP1-PepT1 pathway gene expression, and CDX2/SP1 mediated the regulation of PepT1 by tributyrin.

## 4. Discussion

Tributyrin has been investigated to play similar roles to those of butyrate to affect the growth of animals [19,20]. Butyrate induced the mRNA expression of di/tri-peptide transporter PepT1 and also enhanced its transporting activity [30]. However, the answer to the question of how tributyrin affects the growth of grass carp and regulates the expression of PepT1 in the intestine of juvenile grass carp is still elusive.

In this study, tributyrin was supplemented to feed and its effect on the growth performance of grass carp was examined. We found that appropriate supplementation of tributyrin promoted the growth of grass carp, and the 100% survival rate demonstrated that the tributyrin supplemental concentrations we used in this study showed no toxicity to the fish. These suggested that tributyrin had a similar function as that of sodium butyrate on the growth of fish [8,9]. In this study, although the SGR of moderate tributyrin supplementation groups was promoted significantly compared with the control groups, it was still obviously lower than those of several previous studies. Generally, SGR was related to several factors, including initial weight, feeding model, time period, and other factors. The grass carp with light initial weight (about 0.36 g–5.0 g) exhibited much higher SGR than those of large-initial-size ones during a similar or equal feeding period [42,43,44,45,46]. The mean initial weight of grass carp used in our study was more than 40 g, which would be one factor affecting the SGR. In addition, the feeding period of the grass carp in a previous study was always 8 weeks; a 4-week feeding period is relatively short to fully exhibit the effects of the feed. However, the low SGR of this study was not unique with 4-week feeding period; a previous study with a 4-week grass carp feeding period showed similar SGR to that of this study [47]. Moreover, 4-week feedings were also conducted in previous studies to demonstrate the effect of dietary inclusion on aquatic animal growth [48,49,50]. Despite the low SGR, we still found the significant promotion effect of a tributyrin inclusion diet on the growth of juvenile grass carp compared with that of the control diet. Consequently, it is complicated to elucidate the influence on grass carp SGR and other growth parameters. The comparisons we made above were not conducted in the same conditions, so further specific studies with comprehensive designs are needed to extensively demonstrate TB influence on growth performance, including SGR, of juvenile grass carp. In spite of that above, rare reports of tributyrin applications have been shown in aquatic animals, and our study may be the newest update of tributyrin application in freshwater fish to promote growth. Further study is needed for the application evaluation of TB in grass carp feed. 

PepT1 is the transporter of di/tri- peptide in the intestines. Over the past two decades, people have gradually improved their understandings of protein nutrition and realized that oligopeptide nutrition is a pivotal part of protein nutrition due to its faster speed and greater quantity of transportation by PepT1 compared with amino acid nutrition in animals [51,52]. So, it is significant for oligopeptide transportation to regulate the expression of PepT1. Several studies have investigated that butyrate could influence growth through their regulation on PepT1. It has been evidenced that butyrate induced the increase of hPepT1 expression via its activation on CDX2 transcription activity to the PepT1 promoter [30], while other studies indicated that CDX2 regulated PepT1 via SP1, as the hPepT1 promoter lacked the TATA-box of the CDX2 binding sites but contained some GC-rich sites, which were supposed to bind with the transcription factor Sp1 [37]. In addition, butyrate or sodium butyrate would influence the intestine-related or signal pathway gene expression through its regulation of SP1 in humans [53]. In terms of fish, butyrate was shown to regulate PepT1, possibly through CDX2 in triploid fish [31]. Sodium butyrate upregulated grass carp PepT1 expression in vivo and in vitro [36]. Always, tributyrin, the precursor of butyrate, showed similar functions to those of butyrate. In this study, the results showed that tributyrin played a similar role in promoting the intestine gene CDX2, SP1 and PepT1 expression as that of butyrate (Figure 1 and Figure 2), although the promotion function of tributyrin is not dose-dependent according to its influence on growth performance (Table 1) [31,33]. The appropriate supplementation level or treatment concentration promoted the growth and the expression levels of the oligopeptide-transportation-related genes of grass carp. However, sodium butyrate has been investigated to increase human PepT1 (hPepT1) promoter activity to promote its expression in a dose-dependent manner [30]. The difference in the influence model between butyrate and tributyrin may be due to the following reasons. One is the different species that they worked in: humans represent high-vertebrate animals, while grass carps are low vertebrates. There are huge differences between high- and low-vertebrate animals in nutrient metabolism, even of the same nutrients. Another reason would be the differences between tributyrin and butyrate in the process and mechanism of action in the intestines, although the specific regulation pathway of TB on PepT1 in grass carp is still elusive to date. 

To further investigate the specific regulation pathway, the CDX2/SP1-mediating tributyrin regulation of the expression of PepT1 was hypothesized according to previous studies and our results. Before that, GS, which has been demonstrated to inhibit the expression of CDX2 via NF-κB, and MA, an inhibitor of SP1, were examined for their inhibition of the expression of CDX2 and SP1 in grass carp, respectively. The results showed that GS exhibited an inhibition effect on the expression of NF-κB and CDX2, similar to previous studies [40], while its inhibition effect on CDX2 was not too obvious compared with its effect on NF-κB (Figure 3). This would be due to its indirect inhibition on CDX2. MA showed an obvious inhibition effect to grass carp SP1 (Figure 4). Subsequently, independent or combination injections of TB with GS or MA were conducted. The results showed that tributyrin boosted the expression of CDX2, SP1, and PepT1, and when tributyrin was intraperitoneally injected with GS or MA, the expressions of these genes were blocked compared with those of the individual tributyrin injection group (Figure 5). These results suggested that tributyrin stimulated the expressions of CDX2, SP1, and PepT1, and the altered expressions of CDX2 and SP1 would affect tributyrin regulation of PepT1. Furthermore, the results found that GS would inhibit the expression of SP1, and MA would inhibit the expression of CDX2. A previous study showed that CDX2 regulated PepT1 via the assistance of SP1 in humans [54], while another study reported that CDX2 interacted with SP1 and could directly bind to the promoter of PepT1 to regulate its expression in grass carp [38]. These results indicate that the regulation of PepT1 by CDX2/SP1 was different between higher vertebrates and lower vertebrates. Additionally, the results of GS and MA injection trails exhibited that CDX2 and SP1 could interplay. They may work together in other signal pathways with regulation and feedback regulation in grass carp. However, these hypotheses need further study.

## 5. Conclusions

In this study, we verified that appropriate levels of TB significantly promoted the growth performance and the expression levels of oligopeptide-transportation-related genes (CDX2, SP1, and PepT1) of juvenile grass carp. The inhibitors GS and MA were investigated to have a similar inhibition effect on CDX2 and SP1, respectively, in grass carp to those in other species. TB boosted the expression levels of CDX2, SP1, and PepT1, and its positive regulation of PepT1 was disturbed when CDX2 or SP1 expression was inhibited. These results suggest that CDX2/SP1 mediated the TB regulation of PepT1. The findings of this study may help us to better understand the functions of TB on growth and PepT1 regulation, which could be modulated by dietary TB through the CDX2/SP1-PepT1 pathway in juvenile grass carp. 

## Figures and Tables

**Table 1:** Diet formulation and proximate composition of the experimental diet (% in dry weight).

Ingredients	Percentage Dry Weight (%)
Wheat flour	8.00
Starch	30.00
Soybean meal	32.00
Fish meal ^1^	18.50
Soybean oil	2.50
Fish oil	3.00
Choline chloride	0.5
Monocalcium phosphate [Ca(H_2_PO_4_)_2_]	1.0
Chromium hemitrioxide (Cr_2_O_3_)	0.5
Methyl cellulose	2.00
Mineral Premix ^2^	2.00
Total	100.00
Proximate composition (% dry matter)	
Crude protein	30.26
Crude lipid	8.00
Ash	5.35
Moisture	6.15
Carbohydrate	50.24
Gross energy (MJ/kg)	19.00

^1^ Fishmeal: purchased from American Seafood Company, USA. ^2^ Mineral premix (mg/kg diet): NaCl, 500.0; MgSO_4_·7H_2_O, 8155.6; NaH_2_PO_4_·2H_2_O, 12,500.0; KH_2_PO_4_, 16,000.0; CaH_2_PO_4_·2H_2_O, 7650.6; FeSO_4_·7H_2_O, 2286.2; C_6_H_10_CaO_6_·5H_2_O, 1750.0; ZnSO_4_·7H_2_O, 178.0; MnSO_4_·H_2_O, 61.4; CuSO_4_·5H_2_O, 15.5; CoSO_4_·7H_2_O, 0.91; KI, 1.5; Na_2_SeO_3_, 0.60; and corn starch, 899.7.

**Table 2:** Sequences of designed primers used in this study.

Primer	Accession Number	Sequence (5’ to 3’)
PepT1-qPCR-F	JN088166	TGCTCTTGTTGTGTTCATCG
PepT1-qPCR-R		CTCTCTCTTGGGGTATTGCTT
CDX2-qPCR-F	KC748025	TTTGTAACCGCACCTCC
CDX2-qPCR-R		AGTTCCTGGCCCATAAGT
Sp1-qPCR-F	KY081668	AGTGACCCCAGTAAGAAGAAGCA
Sp1-qPCR-R		CAAGTGTGCCCGCAGATG
NF-κB-qPCR-F	KY129991	GCGTCTATGCTTCCAGATTTACC
NF-κB-qPCR-R		ACTGCCACTGTTCTTGTTCACC
β-Actin-F	M25013	CCTTCTTGGGTATGGAGTCTTG
β-Actin-R		AGAGTATTTACGCTCAGGTGGG

Note: “F” represents the forward primer; “R” represents the reverse primer.

**Table 3:** Effects of different levels of dietary TB on growth performance parameters of grass carp.

Tributyrin Levels	IBW ^1^ (g)	FBW ^2^ (g)	SR ^3^ (%)	WGR ^4^ (%)	SGR ^5^ (%/d)
0 g/kg	43.20 ± 0.088	61.70 ± 3.217 ^a^	100.0 ± 0	42.82 ± 0.072 ^a^	0.64 ± 0.001 ^a^
0.5 g/kg	43.30 ± 0.018	67.80 ± 1.096 ^b^	100.0 ± 0	56.58 ± 0.026 ^b^	0.80 ± 0 ^b^
1.0 g/kg	43.20 ± 0.088	75.30 ± 16.29 ^c^	100.0 ± 0	74.31 ± 0.151 ^c^	0.99 ± 0.002 ^b^
1.5 g/kg	43.60 ± 0.194	60.20 ± 4.278 ^a^	100.0 ± 0	38.07 ± 0.105 ^a^	0.58 ± 0.001 ^a^

Note: Values are Means ± SE (*n* = 4 for each treatment with total 80 fish); Tukey’s multiple range test was performed, and different letters represent significant differences (*p* < 0.05); abbreviations: ^1^ IBW, initial body weight; ^2^ FBW, final body weight; ^3^ SR, survival rate; ^4^ WGR, weight gain rate; ^5^ and SGR, specific growth rate.

## Data Availability

The datasets used and/or analyzed during the current study are available from the corresponding author on reasonable request.
